# A novel enucleation– exenteration approach of the equine eye via the supraorbital fossa: an experimental and clinical study in donkeys (*Equus asinus)*

**DOI:** 10.3389/fvets.2024.1379151

**Published:** 2024-06-14

**Authors:** Mohamed Marzok, Adel I. Almubarak, Mohamed Nazih, Thnian A. Al-thnaian, Khalid Alkhodair, Mohamed El-Sherif

**Affiliations:** ^1^Department of Clinical Sciences, College of Veterinary Medicine, King Faisal University, Al-Ahsa, Saudi Arabia; ^2^Department of Surgery, Faculty of Veterinary Medicine, Kafrelsheikh University, Kafrelsheikh, Egypt; ^3^Department of Anatomy, Faculty of Veterinary Medicine, New Valley University, Al-Kharga, Egypt; ^4^Department of Anatomy, College of Veterinary Medicine, King Faisal University, Al-Ahsa, Saudi Arabia; ^5^Department of Surgery, Faculty of Veterinary Medicine, New Valley University, Al-Kharga, Egypt

**Keywords:** donkey, enucleation, exenteration, supraorbital fossa, novel technique

## Abstract

This study investigated the description and feasibility of a surgical procedure for enucleation–exenteration of the equine eye via the supraorbital fossa. A preliminary study was performed on both eyes of four cadaveric heads of native breed donkeys (*Equus asinus*) to describe the surgical anatomy and demonstrate a new supraorbital enucleation surgical approach. For the clinical study, eight donkeys were admitted for unilateral enucleation. All procedures were performed in a lateral recumbent position under the influence of inhalation anesthesia in combination with a retrobulbar nerve block. A semi-circular incision was made in the skin and fascia of the supraorbital fossa to gain access into the orbital cavity, after which the periorbital fat was dissected and removed. Bleeding was controlled by electrocautery and large blood vessels were ligated, then the eyeball was dissected sharply and freed from its bony attachment. The procedure was successfully accomplished in all clinical cases, and no significant complications occurred during or throughout the postoperative follow-up period. The initial results suggest the feasibility and safety of the supraorbital enucleation technique described in this study for equine eye enucleation. This new technique seems promising due to its feasibility, safety, and positive outcomes observed in both cadaveric and clinical studies.

## 1 Introduction

Enucleation refers to the removal of the eye, conjunctiva, third eyelid, third eyelid gland, and lacrimal gland (1–3) and a common surgical procedure that may be necessary to alleviate pain, remove a tumor, hematoma, or control severe corneal infection or endophthalmitis (4–6). Two distinct surgical techniques for enucleation–exenteration of the eyeball, the transpalpebral (3, 7), and the transconjunctival (8), are widely performed in standing horses under sedation and loco-regional analgesia or general anesthesia (9). Transpalpebral enucleation entails removing the bulbar fornix, palpebral conjunctiva, third eyelid along with its gland, and the globe, and dissecting deeply to the extraocular muscle cone (10). Subconjunctival enucleation involves removing the globe from the Tenon capsule via a peritomy incision (11). These techniques, while effective, can result in general complications such as hemorrhage, infection, wound dehiscence, orbital pneumatosis, inclusion cyst, and delayed healing (12–14). The enucleation procedure can be due to several factors, including the mobility of the eye, limited exposure of the orbital cavity, and the presence of large retrobulbar masses (9, 10, 15). Common serious complications associated with the procedure are the failure to remove all ocular and orbital secreting tissue and damage of optic chiasm due to forced traction of the globe that leads to blindness of the contralateral eye (3, 9). Furthermore, conventional enucleation procedures are known for being bloody surgeries, often accompanied by reported varying degrees of intraoperative bleeding (16, 17). For that reason, the use of blood-sealing devices such as monopolar and bipolar electrocautery was reported to minimize these complications (1, 18). While the use of these devices is recommended, their availability may pose an obstacle for some practitioners, thus adding to the limitations of traditional procedures. Enucleation followed by orbital implant placement is a viable cosmetic procedure for irreversibly blind eyes, with reported lower complication rates (19). Extrusion and implant rotation are reported as complications (20, 21). Average implant failure was reported in horses due to large orbital cavities (20). Diminishing orbital infection and application of large flattened orbital implants followed by tarsal raphe were recommended to address the failure (8). In this study, our aim was to investigate and propose a new method for the surgical removal of the equine eye through the supraorbital fossa. The primary objectives were to address the limitations of traditional techniques and to evaluate the feasibility, effectiveness, and potential advantages of this novel approach compared to traditional transpalpebral and transconjunctival enucleation techniques.

## 2 Materials and methods

### 2.1 Animal ethics statement

This experiment was reviewed and approved by the Research Ethics Committee (REC) of King Faisal University, KSA (Approval No. KFU-REC/2023-EA000823).

### 2.2 Experimental plan

To gain an understanding of the surgical anatomy of the equine eye and to develop the supraorbital enucleation–exenteration technique, a two-phase study was conducted. Phase 1 included a preliminary study on four cadaveric heads of native-breed donkeys. An anatomical dissection of one eye (the right eye) was performed to describe the surgical anatomy of the ocular structures. Uniform and systemic anatomical dissection is performed using the same tools and dissection protocol by a single anatomist. Next, an experimental surgical procedure was performed on the other eye (left eye) of the cadaver to evaluate the feasibility of our proposed technique. The cadaveric study was performed uniformly by the same surgeon with the same tools and standardized protocol, establishing clear procedural steps, anatomical landmarks, and instrument usage protocols. Based on the results of this initial study, we then conducted phase 2 which involved a clinical study on eight adult donkeys with variant ocular pathologies to further assess the safety and efficacy of our technique in live animals. Standardized preoperative preparation protocols were followed for all experimental subjects including preoperative fasting period, pre-anesthetic medication administration, and surgical site preparation protocol. Anesthesia protocols were standardized for all animals including the anesthetic agent, dose, and application method. Surgical instruments were also the same, and the surgical procedures were performed by the same surgeon using a standardized approach in all animals. Postoperative outcomes, wound healing, and complications were assessed by individuals who were blinded to the surgical method.

#### 2.2.1 Phase 1—Surgical anatomy and experimental surgery

Four fresh heads of an adult native donkey breed (*Equus asinus*) were obtained after euthanasia by the local veterinary authority due to old untreated complete open tibial fracture, comminuted second phalanx fracture, radial nerve paralysis, and obturator nerve paralysis. The right orbital and supraorbital areas were clipped and thoroughly cleaned. Sharp and blunt anatomical dissection of the eye and orbital structures were performed, and anatomical data were recorded.

**Figure 1:**
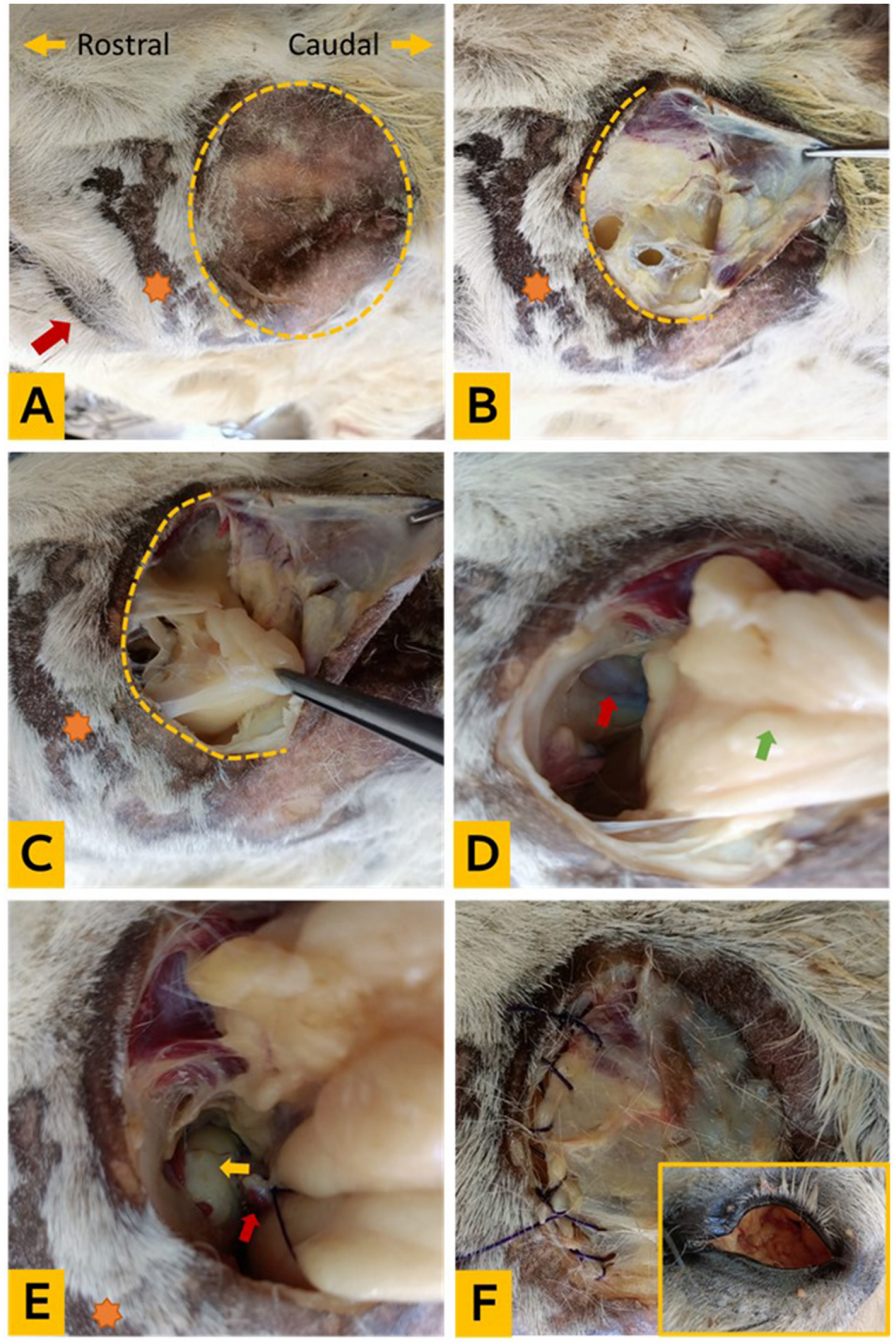
Experimental enucleation technique via supraorbital fossa. **(A)** The orientation of the cadaver head and clipping of the supraorbital fossa (yellow dotted circle), the orange star indicates the ciliary process, and the red arrow points to the eye; **(B)** exposure of the subcutaneous fascia; **(C)** periorbital fat; **(D)** identification of the Tenon capsule, optic nerve, and ophthalmic vessels (red arrow); and the orbital fat (green arrow); **(E)** ligation of the optic nerve and vessels (red arrow) and eyeball (yellow arrow); **(F)** removal of the entire orbital socket content and suturing of the fascia (the in frame photo showing the orbital cavity after removal of its contents).

On the same heads, the left supraorbital fossae and orbital areas were clipped and disinfected. A semicircular incision of the skin and fascia was made by a scalpel at the rim of the orbital fossa with the free ends directed rostrally. Following, a sharp dissection was performed to make a skin flap. Sharp and blunt dissection and extraction of the periorbital fat were then performed. A sharp dissection of the Tenon capsule was performed down to the extraocular muscles and eyeball to free it from the bony orbital cavity. Careful dissection was carried out with a 20-cm curved dissecting scissors to expose the optic nerve and vein and the large ophthalmic vessel. A needle driver was utilized to pass a 2-0 polyglactin suture *(Egycryl, Taisier-Med, Egypt)* and subsequently ligate it with buried knots before severing the ligated structures. The freed eyeball was then dissected from the conjunctiva and eyelids using a scalpel and curved Metzenbaum scissors. The eyeball was removed and the orbital cavity was irrigated with saline and antiseptic solution. The supraorbital fascia was sutured with number 1-0 polyglactin *(Egycryl, Taisier-Med, Egypt)* and skin opposed with number 0 silk sutures *(Egysilk, Taisier-Med, Egypt)* in a simple interrupted pattern. The surgery method for supraorbital enucleation is depicted in Figures 1A–F.

#### 2.2.2 Phase 2—Supraorbital enucleation procedure

**Figure 2:**
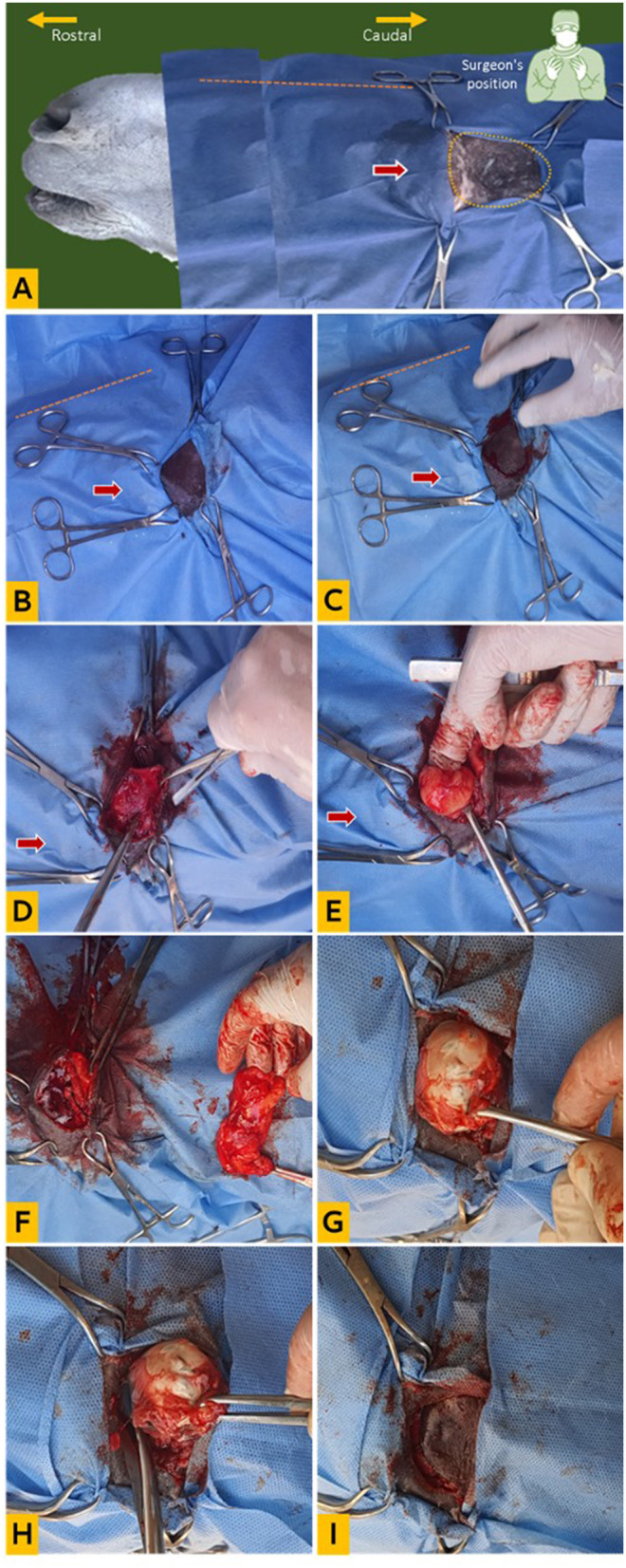
Clinical enucleation technique via supraorbital fossa. **(A)** Head orientation for surgery, the yellow dotted circle indicates the supraorbital fossa, the red arrow points to the eye (draped), and the orange dotted line indicates the nasal bridge; **(B)** preparation of surgical site (double draped); **(C)** creation of a **(C)**-shaped skin incision on the supraorbital rim; **(D)** dissection of the fascia; **(E)** exposure of the orbital fat; **(F)** dissection and removal of the orbital fat; **(G)** dissection of the posterior attachments of the eyeball; **(H)** dissection of the eyeball from the palpebral attachment; **(I)** replacement of the skin flap for suturing.

This procedure was conducted on eight local breed donkeys (*Equus asinus*) admitted to the Veterinary Teaching Hospital of the College of Veterinary Medicine, King Faisal University. Each of the eight donkeys was suffering from either unilateral perforated eye and damage of the eye lens (5 donkeys), large bulbar conjunctival mass (1 donkey), or chronic ulcerative deteriorating keratitis (2 donkeys). The donkeys were all adult males with a mean age of 7 ± 1.5 years and a mean body weight of 153 ± 11.4 kg. Prior to surgery, food was withheld for 12 h but water was allowed. A 14-gauge intravenous catheter was placed in the jugular vein of the same side of the affected eye. Amoxicillin trihydrate 10 mg/kg (*Amoxykel 15%, Kela, Belgium*) was administered intramuscularly on the day of surgery. Immediately before the surgery, flunixin meglumine was administered intravenously at a dose of 1 mg/kg body weight (*Flunixin injection, Norbrook, Ireland*). The eight donkeys were generally anesthetized according to the standard protocol for equine general anesthesia recognized by the regulations of the hospital. Xylazine (*Xyla, InterCheme, Holland*) was administered at a dose of 1 mg/kg intravenously, and anesthesia then induced by administration of 0.05 mg/kg diazepam *(Dazam, Richmond Vet Pharma, Argentina)* and 2.5 mg/kg ketamine *(Ketaset; Zoetis)* given intravenously, and the donkeys were intubated with size 25 cuffed ETT and maintained on sevoflurane (Sevorane; Abbot, UK) vaporized in 100% oxygen, using a large animal circle system. During the surgery, each case had intravenous fluids (isotonic saline solution, 10 ml/kg/day), and indirect arterial blood pressure was monitored with a vital monitor. Retrobulbar administration of 10 ml lidocaine 2% *(Lidocaine, Pharmaceutical Solutions Industry, Jeddah, KSA)* through a 22-gauge, 90 mm spinal needle placed perpendicular to the skin in the deepest part of the orbital fossa approximately 0.5 cm caudal to the posterior aspect of the boney dorsal orbital rim and advanced caudal to the globe until it reaches the retrobulbar orbital cone. Afterward, each donkey was placed in lateral recumbency with the affected eye positioned upward. The orbital and supraorbital areas were clipped, disinfected, and prepared for surgery. A double-layer four-corner drape was placed over the entire head, and the orbital fossa was uncovered. The area below the drape and the eye was covered with a layer of povidone iodine 10% and the lower drape layer isolated the eye from the head. A semi-circular incision in the skin and fascia was performed at the rim of the orbital fossa with a scalpel, and the free end of the incised skin was directed rostrally. A sharp dissection with scissors was performed, and the skin flap was flipped caudally to expose the periorbital fat and orbital structures. By using a long curved Metzenbaum dissecting scissors, the periorbital fat was excised. Bleeding from orbital fat pad vasculature was controlled by a monopolar electrocautery device followed by irrigation of the orbit with isotonic saline solution to remove the blood and provide better visualization. The globe and dorsal extraocular muscles were visible. Irrigation of the orbital cavity with saline and suction was performed to enhance the visibility of structures and to detect bleeding spots. The surgeon used his free hand to gently manipulate the eyeball through the palpebral fissure, facilitating dissection of the bulbar fornix and palpebral conjunctiva combined with the transpalpebral division. The bleeding was controlled through electrocautery of the source vessels. The large blood vessels and optic nerve were ligated with a 2-0 polyglactin suture (*Surgcol, Supersurgmed LTD, India)*. Further dissection of the ventral extraocular muscles continued to free the entire globe except for the anterior conjunctival portion. Blunt dissection with long Metzenbaum curved scissors was used to separate the bulbar conjunctiva from the sclera. The dissection continued circumferentially until the entire globe was completely freed. The eyeball was removed, and the orbital socket was backed with sterile gauze tinged with antiseptic iodine solution 10%, the supraorbital fascia was approximated using 1/0 polyglactin (*Surgcol, Supersurgmed LTD, India)*, and the skin was closed with interrupted sutures using No. 0 polypropylene (*Surgprop, Supersurgmed LTD, India)*. The surgical technique for supraorbital enucleation is illustrated in Figures 2A–I.

#### 2.2.3 Long-term management, monitoring, and outcome

After surgery, the donkeys were monitored intensively, and given 1,500 IU of antitetanic serum *(Tetanus antitoxin, Pasteur Lab.)*. The antimicrobial cover, Amoxicillin trihydrate *(Amoxykel, Kela, Belgium)*, at 10 mg/kg intramuscularly, and the anti-inflammatory analgesic flunixin meglumine *(Finadyne, MSD, USA)* at 1.1 mg/kg intravenously, were continued for 5 successive days. A short-term follow-up was conducted by the surgeon for 15 days until the removal of the skin sutures. Complications were recorded. The donkeys were discharged, and follow-up care was coordinated with local veterinarians, which included subsequent monitoring via telephone calls.

#### 2.2.4 Surgery assessment

The evaluation criteria included feasibility, procedural duration, safety, and complications. Feasibility refers to the assessment of its practicality, viability, and potential for successful execution. It involves considering factors such as technical requirements, available resources, and the likelihood of achieving the desired outcomes while minimizing risks and complications. Procedural duration was conducted from the initial skin incision to the last suture of the skin in minutes. Safety refers to the extent to which the intervention minimizes risks and contrary outcomes. It involves potential complications, adherence to established protocols, and the use of appropriate techniques and precautions. Intraoperatively, assessment criteria included adequacy of anesthesia, intensity of bleeding, feasibility, and invasiveness of the procedure. Postoperatively, evaluations involved monitoring for bleeding, swelling, infection, inflammation, the integrity of wound healing, and cosmetic appearance.

## 3 Results

### 3.1 Surgical anatomy

**Figure 3:**
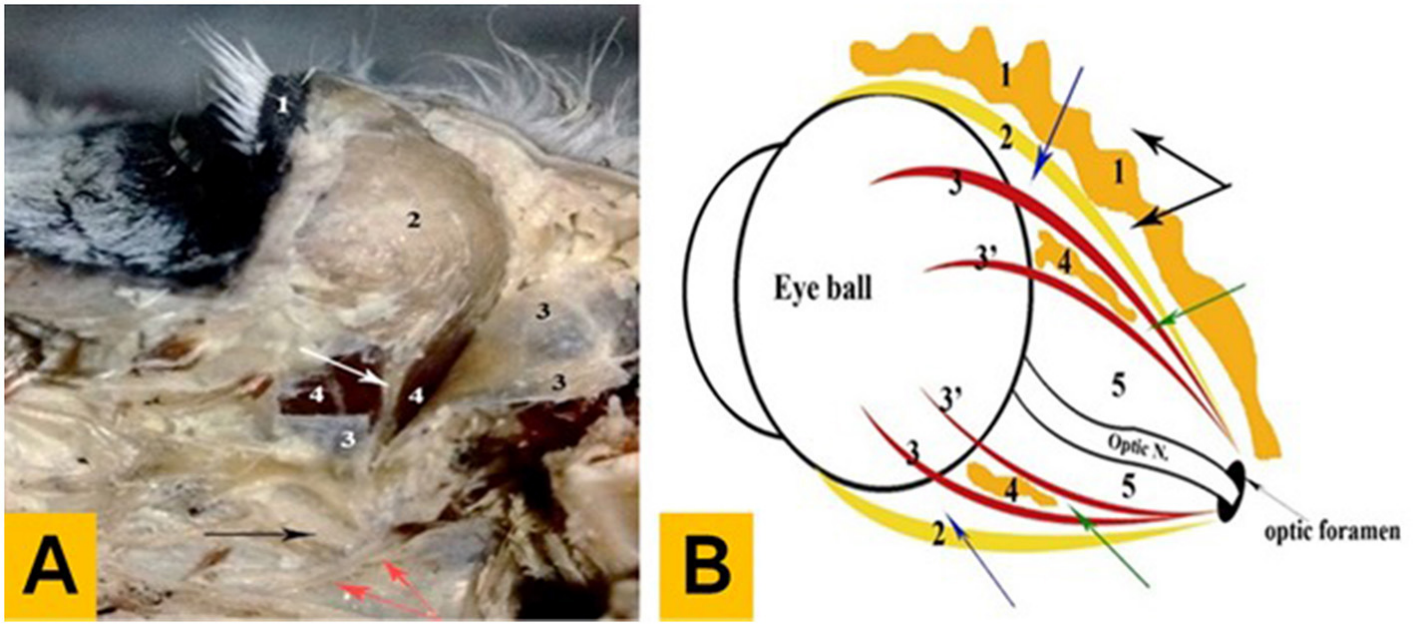
Anatomical findings of the eye in donkeys. **(A)** Photograph showing the deep dissection of the orbital cavity (left lateral aspect): 1—upper eyelid, 2—lacrimal gland, 3—Tenon capsule (open), 4—muscular capsule (outer sublayer), the white arrow indicates the lacrimal nerve and artery, the black arrow indicates the maxillary nerve, and the red arrows indicate the pterygoid crest. **(B)** Diagram showing the arrangement pattern of the orbital fasciae and intervening spaces: 1—external periorbital fascia, 2—Tenon capsule, 3—the muscular capsule (outer sublayer), 3'—the muscular capsule (inner sublayer), 4—internal periorbital fat, and 5—inner orbital space, the black arrow indicates the periorbital space, the blue arrow indicates the outer orbital space, and the green arrow indicates the middle orbital space.

The orbital fossa is a part of the orbital region and can be considered its superior entrance. This fossa is located rostrally from the caudal border of the zygomaticofrontal process and reaches dorsally to the orbital line. It is restricted ventrolateral by the orbital crest and zygomaticoorbital process and continues in a spacious manner on the parietal and squamous parts of the temporal bones. The temporal fossa is covered by skin and contains fatty masses, orbital fasciae, and the eye and its adnexa. The orbital fasciae consist of a double-walled envelope with a fibrous Tenon capsule and a muscular inner layer. The Tenon capsule encloses the extrinsic ocular muscles, eye, and optic nerve attached to the fibrous ring around the optic foramen. The inner sublayer of the muscular capsule includes the recti and oblique muscles, while the outer sublayer contains the retractor bulbi muscle. The inner orbital space is occupied by the internal periorbital fat and contains the optic nerve, bulbar innervation, and terminal branches of the external ophthalmic artery. The surgical anatomy of the eye is shown in Figures 3A, B.

### 3.2 Supraorbital enucleation procedure

The procedure was successfully accomplished in all eight cases, and no significant intraoperative complications were encountered. The technique enabled the complete removal of the eyeball, nictitating membrane and gland, lacrimal gland, and all periorbital structures within the orbital cavity while the eyelids and palpebral conjunctiva remained intact. The technique was feasible and ordinary general surgical tools and resources were sufficient to perform the surgery. The mean procedural time was 46.25 ± 1.38 min. Bleeding was a notable intraoperative complication, observed after the dissection of the periorbital fat and during the separation of the periorbital attachments. It was successfully controlled using electrocautery. The ophthalmic and other deep-seated blood vessels, along with the optic nerve, were ligated with moderate effort, involving the use of burying knots. The use of the surgeon's free hand for fixation and reduction of the eyeball facilitated dissection and reduced the required time. During the 15 days of the postoperative follow-up period, none of the operated donkeys showed significant complications. Minimal hemorrhage and swelling were seen postoperatively in all donkeys and lasted < 48 h. No adverse effects were seen from the anesthesia used. None of the donkeys developed blindness in the contralateral eye or any signs of neuropathy. Normal behavior, appetite, and physiological parameters were recorded. The supraorbital wounds were found covered with healthy granulation tissue within 17.25 ± 1.28 days. The extent of orbital cavity granulation varied among the operated donkeys, as documented by the referring veterinarians during the follow-up period. However, the formed granulation tissue was reported to exhibit characteristics of fresh bleeds upon touch and not excessive, displaying a pink color indicating a healthy appearance. The extent of granulation ranged from approximately a third to half of the orbital socket. No significant long-term complications were reported by referring veterinarians or the donkeys' owners for three successive months after surgery.

## 4 Discussion

Enucleation is a surgical procedure designed for the removal of the eye to achieve two main goals: removal of the diseased eye with minimal complications (3, 8, 22) and achieving a better cosmetic appearance (5, 23, 24). Most current approaches for enucleation in horses include two main methods, the transpalpebral and transconjunctival approaches (3, 7, 8). Herein, we described a new technique to approach the entire orbital cavity content through the supraorbital cavity in donkeys. The temporal fossa, also known as the supraorbital or orbital fossa, is a depression in the skull bound by the parietal, zygomatic, and frontal bones. In addition to the temporalis muscle, it contains periorbital fat and is lined dorsally with a skin layer. In older animals, the peri-orbital fat is reduced and the fossa became more distinct (25). The temporal fossa in equine is larger than other species and typically used to employ retrobulbar block of the eye (26). The results of the current study indicated that the orbital fossa was observed to be a large enough and secure entry point to the retrobulbar structures. After the removal of the orbital fat pad, which is advised with enucleation, the ocular structures were readily visible, and suitable space for surgery was provided. Complications of enucleation procedures are due to bleeding, inadequate removal of the orbital structures, and subsequent hematoma, infection, cysts, emphysema, or acute inflammation (2). For that reason, the authors reported the use of advanced bleeding control devices (1) and emphasized removal of all orbital structures (5). Our present approach facilitated access and achievement of the previous recommendations. The surgical approach from a window in the orbital fossa described in the current report enabled the use of a monopolar electrocautery device to control bleeding, ligation of large vessels, and removal of entire orbital structures. There was no need for a special bleeding control clamp as reported by Pollock and Russell (3). Other serious complications include damage of the optic chiasm, subsequent meningitis, or blindness of the contralateral eye (2, 3). In our opinion, the current technique offers a safer cut of the optic nerve without forced traction of the eyeball, hence reducing the related complications. Moreover, creating a dorsal window to the orbital cavity enables the surgeon to extensively irrigate and rinse the wound and decrease the dependent infection or inflammatory reactions. From a cosmetic perspective, the sunken ocular socket is a common distortion reported after enucleation procedures (5, 23). Although we are working on a relevant study, our hypothesis posits that the implantation of an orbital prosthesis through a surgically created supraorbital window to occupy the entire orbital cavity would yield enhanced cosmetic aesthetics and maintain the normal size of the ocular socket. Another noteworthy aspect of cosmetic significance in the present approach lies in the preservation of structure and functionality of both the eyelids and eyelashes, which gives an acceptable postoperative appearance. The notable intraoperative complication encountered in this approach, as in others, was bleeding (22). However, our current study observed improved bleeding management utilizing a monopolar electrode through the suborbital surgical window. Moreover, the mean surgical time recorded in our study was consistent with that reported by Betbeze and Dray (8). Although the authors recommend standing enucleation under sedation and loco-regional anesthesia (2, 8, 27) reporting more safety, profitability, and fewer complications, in the current study, we did not record any anesthesia-related complications. Lateral recumbency offered the surgeon better observation of the surgical field and avoided the non-relevant movement of donkeys during surgery. Subsequent investigations ought to address the limitations inherent in the current study. These include assessing the technique under sedation in standing animals, expanding the study sample, and conducting a comprehensive evaluation of cosmetic appearance post-ocular prosthesis implantation, along with a long-term assessment of associated complications.

## 5 Conclusion

In conclusion, our study adds to the growing body of literature handling the enucleation techniques in equine. While further studies are needed to fully evaluate the long-term safety and efficacy of the supraorbital enucleation technique presented here, our findings suggest that it is an addition to the widely applied conventional procedures and may offer potential advantages for equine eye enucleation.

## Data availability statement

The raw data supporting the conclusions of this article will be made available by the authors, without undue reservation.

## Ethics statement

This experiment was reviewed and approved by the Research Ethics Committee (REC) of King Faisal University, KSA (Approval No. KFU-REC/2023-EA000823). The studies were conducted in accordance with the local legislation and institutional requirements. Written informed consent was obtained from the owners for the participation of their animals in this study.

## Author contributions

MM: Conceptualization, Data curation, Funding acquisition, Investigation, Methodology, Project administration, Resources, Supervision, Validation, Visualization, Writing – original draft, Writing – review & editing. AA: Conceptualization, Funding acquisition, Methodology, Project administration, Validation, Writing – review & editing. MN: Data curation, Formal analysis, Investigation, Methodology, Validation, Visualization, Writing – original draft. TA-t: Writing – original draft, Writing – review & editing, Validation, Methodology, Project administration. KA: Conceptualization, Data curation, Funding acquisition, Investigation, Methodology, Project administration, Resources, Validation, Visualization, Writing – original draft. ME-S: Data curation, Formal analysis, Investigation, Methodology, Software, Validation, Visualization, Writing – original draft, Writing – review & editing.
